# Monitoring trifluridine incorporation in the peripheral blood mononuclear cells of colorectal cancer patients under trifluridine/tipiracil medication

**DOI:** 10.1038/s41598-017-17282-5

**Published:** 2017-12-05

**Authors:** Ryota Nakanishi, Hiroyuki Kitao, Mamoru Kiniwa, Yosuke Morodomi, Makoto Iimori, Junji Kurashige, Masahiko Sugiyama, Yuichiro Nakashima, Hiroshi Saeki, Eiji Oki, Yoshihiko Maehara

**Affiliations:** 1 Department of Surgery and Science, Graduate School of Medical Sciences, Fukuoka, Japan; 2 Department of Molecular Cancer Biology, Graduate School of Pharmaceutical Sciences, Fukuoka, Japan; 3 Innovative Anticancer Strategy for Therapeutics and Diagnosis Group, Innovation Center for Medical Redox Navigation, Kyushu University, Fukuoka, Japan; 4 Taiho Pharmaceutical Co. Ltd.,, Tokushima and Ibaraki, Japan

## Abstract

Trifluridine/tipiracil (TFTD, TAS-102) is an orally administrated anti-cancer drug with efficacy validated for patients with metastatic colorectal cancer (mCRC). Trifluridine (FTD) is an active cytotoxic component of TFTD and mediates the anticancer effect via its incorporation into DNA. However, it has not been examined whether FTD is incorporated into the tissues of patients who received TFTD medication. By detecting FTD incorporation into DNA by a specific antibody, we successfully detected FTD in the bone marrow and spleen cells isolated from FTD-challenged mice as well as human peripheral blood mononuclear cells (PBMCs) activated with phytohemagglutinin-P and exposed to FTD *in vitro*. FTD was also detected in PBMCs isolated from mCRC patients who had administrated TFTD medication. Intriguingly, weekly evaluation of PBMCs from mCRC patients revealed the percentage of FTD-positive PBMCs increased and decreased in parallel with the administration and cessation of TFTD medication, respectively. To our knowledge, this is the first report to detect an active cytotoxic component of a chemotherapeutic drug in clinical specimens using a specific antibody. This technique may enable us to predict the clinical benefits or the adverse effects of TFTD in mCRC patients.

## Introduction

Colorectal cancer is the third most common type of cancer and the leading cause of cancer death worldwide^1,2^. Systemic chemotherapy has greatly improved overall survival or relapse free survival of metastatic colorectal cancer (mCRC) patients^3–6^. The strategy of systemic therapy for mCRC patients has been shifting to a more personalized or precision-based approach. In these cases, molecularly targeting drugs are selected according to information based on specific molecular biomarkers^7,8^. For example, the mutation status of the *RAS* gene is a biomarker to predict the efficacy of anti-epidermal growth factor receptor antibodies^7,8^. Regarding cytotoxic chemotherapy, many biomarkers have been proposed^9^, but most of these have not been used in clinical situations.

Trifluridine/tipiracil (TFTD, TAS-102) is an orally administrated anti-cancer drug. In a placebo-controlled, global phase III trial named RECOURSE, TFTD significantly improved the overall survival (OS) and progression free survival (PFS) of mCRC patients who were not responsive to prior chemotherapy regimens^10^. Trifluridine (FTD) is an active cytotoxic component of TFTD^11,12^. FTD is phosphorylated by thymidine kinase to FTD 5′-monophosphate (FTD-MP). Further phosphorylation of FTD-MP leads to the accumulation of FTD 5′-triphosphate (FTD-TP), which is incorporated into DNA of tumour cells. DNA dysfunction caused by FTD misincorporation mediates the anticancer effect, but the molecular nature of DNA dysfunction has not been identified. Moreover, FTD-MP transiently binds to the active site of thymidylate synthase^13^, which is a key enzyme of the *de novo* synthesis of thymidine triphosphate (dTTP).

Several factors have been suggested as predictors of the TFTD effect^14–18^. Neutropenia, a major adverse event of TFTD^10^, is a predictor of OS in patients receiving TFTD^14,15^. In contrast, the correlation between blood concentrations of FTD and TFTD efficacy have not been shown^14^. In a mouse xenograft model, a significant correlation between the amount of FTD incorporation into tumour DNA and the antitumor effect of FTD was shown^19^. However, this concept cannot be confirmed in clinical settings because there is no method to evaluate the amount of FTD incorporated into DNA obtained from patient specimens. Previously, we found that several anti-bromodeoxyuridine (BrdU) antibodies specifically recognized FTD, both in its bovine serum albumin (BSA)-conjugated form and in its incorporated form of genomic DNA^20^. Furthermore, FTD in a xenograft was also detected by the immunohistochemical staining of paraffin-embedded samples^20^. In this report, we show the successful detection of FTD in the bone marrow cells and spleen cells of FTD-administrated mice. The proportion of the FTD-positive spleen cells increased by FTD administration, but gradually decreased if FTD administration was ceased. We also successfully detected FTD in actively proliferating peripheral blood mononuclear cells (PBMCs) exposed to FTD *in vitro* and in PBMCs isolated from mCRC patients under TFTD medication. Intriguingly, the FTD-positive PBMCs from mCRC patients increased and decreased in parallel with the administration and cessation of TFTD medication, respectively. This detection method makes it possible to monitor active cytotoxic component of chemotherapeutic drug in the clinical specimens and may permit us to predict the clinical benefits or adverse effects of TFTD in mCRC patients.

## Results

### FTD incorporation is detected in the bone marrow cells of FTD-administrated mice

**Figure 1:**
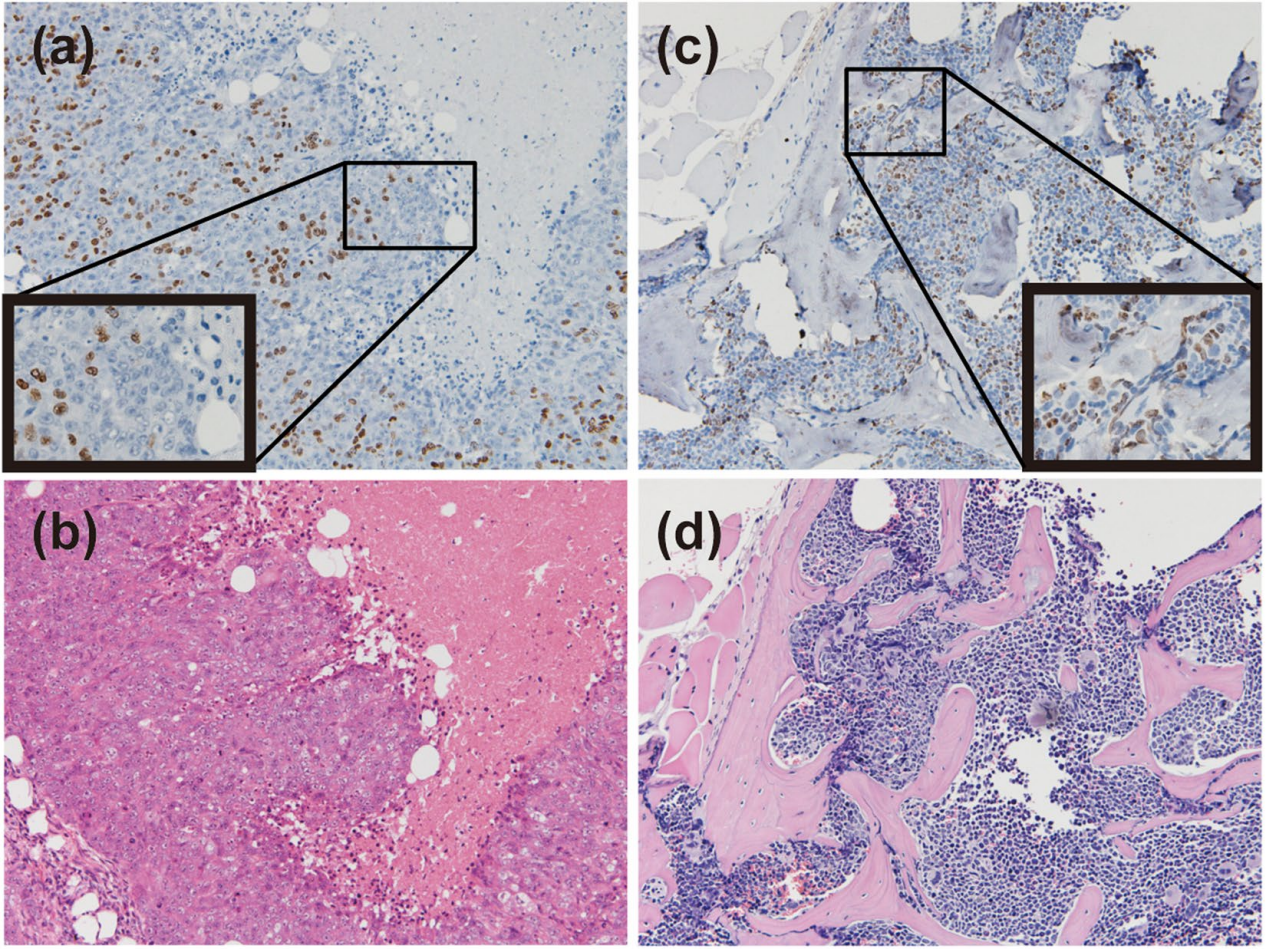
FTD incorporation into xenograft and bone marrow in FTD-administrated BALB/cAJcl-*nu/nu* mice. Immunohistochemical images of FTD incorporation in the xenograft (**a**,**b**) and bone marrow (**c**,**d**) of BALB/cAJcl-*nu/nu* mice. FTD was immunohistochemically stained in the nucleus of both xenograft (**a**) and bone marrow (**c**). HE staining of the xenograft and bone marrow (**b**) and (**d**). Magnification: ×100 (low-power field) and ×400 (inlet, high-power field).

In our previous report, we showed that FTD incorporation into DNA of human tumour cell lines was detected by several anti-BrdU antibodies, even in paraffin-embedded human xenografts of FTD-administrated mice^20^. To determine whether FTD is incorporated into DNA in normal tissues, we prepared paraffin-embedded specimens of normal tissues from FTD-administrated BALB/cAJcl-*nu*/*nu* mice carrying human xenografts and performed immunohistochemical staining of FTD. As previously reported, FTD was detected in tumours in the xenografts (Fig. 1a, haematoxylin-eosin (HE) staining shown in Fig. 1b). In addition, FTD was present in the bone marrow (Fig. 1c, HE staining shown in Fig. 1d), and few FTD-positive cells were observed in the skin, brain or liver (data not shown). This indicates that FTD is incorporated into DNA of tumours as well as some limited normal tissues, such as bone marrow.

### FTD incorporation rate in the spleen cells isolated from FTD-administrated mice fluctuates according to the schedule of FTD administration

**Figure 2:**
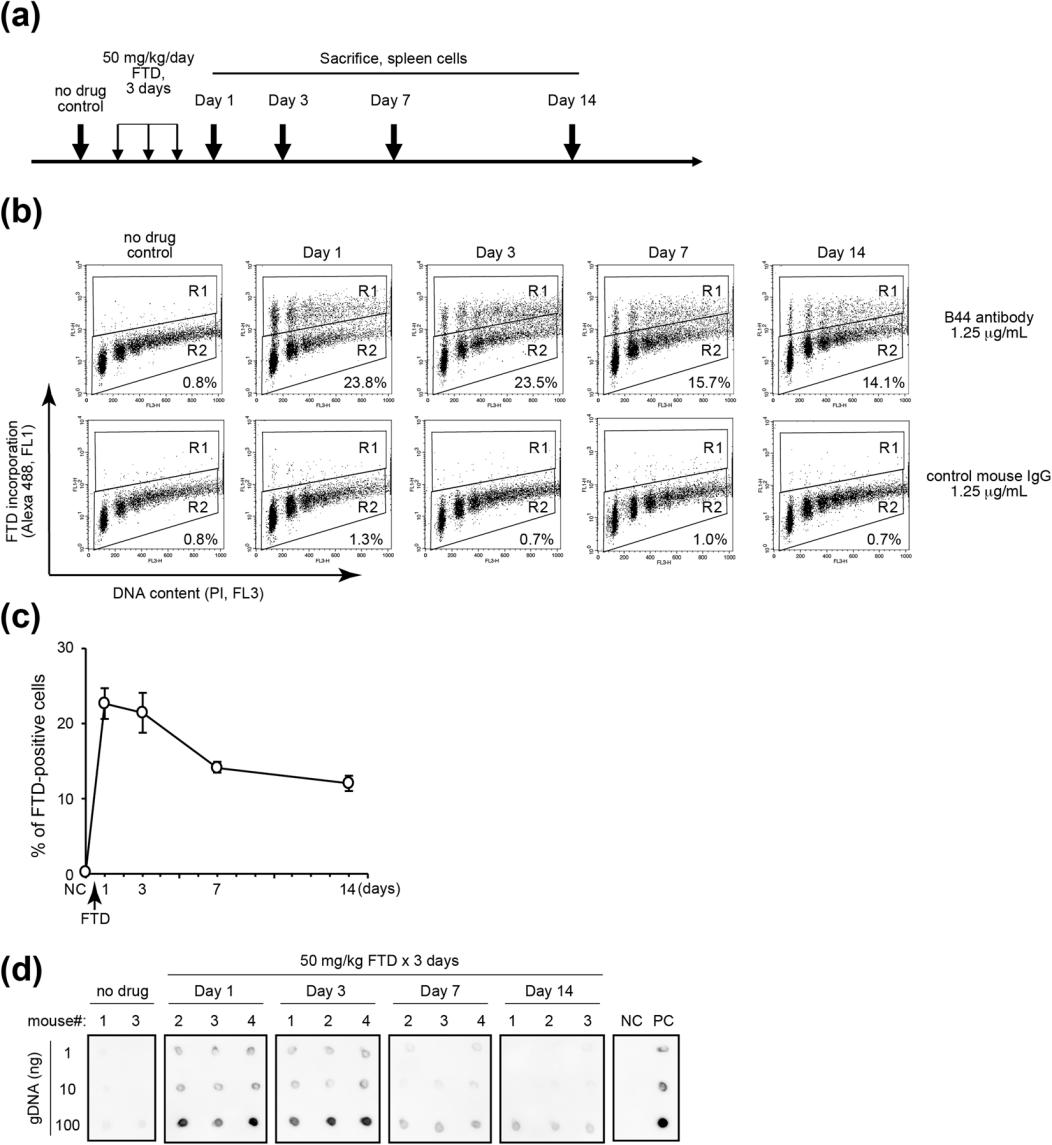
FTD incorporation in the spleen cells of FTD-administrated BALB/cAJcl mice. (**a**) The schedule of FTD administration and sampling. FTD (50 mg/kg) was orally administrated for three days. Day 1 was the day after the last administration of FTD. The spleen was isolated on days 1, 3, 7 and 14. (**b**–**d**) Detection of FTD-positive spleen cells by FACS analysis (**b**,**c**). The samples were incubated with anti-BrdU antibody (**b**; upper) or with control mouse IgG antibody (**b**; lower). R1: FTD-positive region; R2: FTD-negative region. PI, propidium iodide. (**c**) The % of FTD-positive spleen cells. Data are the means ± standard error (n = 4). (**d**) Detection of FTD in DNA of spleen cells from individual mice by dot blot analysis. NC: negative control (genomic DNA from non-treated HCT116 cells); PC: positive control (genomic DNA from HCT116 cells treated with 5 µM FTD for 4 hours). This figure was cropped from the same gel (Supplementary Figure 1).

Efficient FTD incorporation in bone marrow cells might explain adverse effects such as neutropenia, which frequently appear in patients receiving TFTD medication^10^. To determine whether FTD administration affects the incorporation rate of FTD in lymphoid cells, we orally administrated FTD to BALB/cAJcl mice (Fig. 2a) and analysed the FTD incorporation in spleen cells by fluorescence activated cell sorter (FACS) using anti-BrdU antibody B44 (Fig. 2b). The specificity of FTD recognition by the B44 antibody was confirmed by the absence of FTD-positive spleen cells isolated from mice without FTD treatment (Fig. 2b, upper left). The threshold was determined by the value of signal intensity of FTD incorporation when B44 antibody was replaced with control mouse IgG antibody (Fig. 2b, dot plots in lower row). At Day 1 after 3 consecutive twice-daily administrations of FTD (50 mg/kg/day), more than 20% of spleen cells were FTD-positive (Fig. 2b,c). The percentage of FTD-positive spleen cells gradually decreased to about 10% at Day 14 (Fig. 2b,c). This acute increase and gradual decrease of FTD-positive spleen cells was recapitulated by dot blot analysis of genomic DNA purified from spleen cells (Fig. 2d). Collectively, our data indicate that FTD is incorporated into DNA of lymphoid cells and that the proportion of FTD-positive lymphoid cells gradually decreases after the cessation of FTD administration.

### FTD is incorporated into DNA of human PBMCs activated with phytohaemagglutinin-P and exposed to FTD *in vitro*

**Figure 3:**
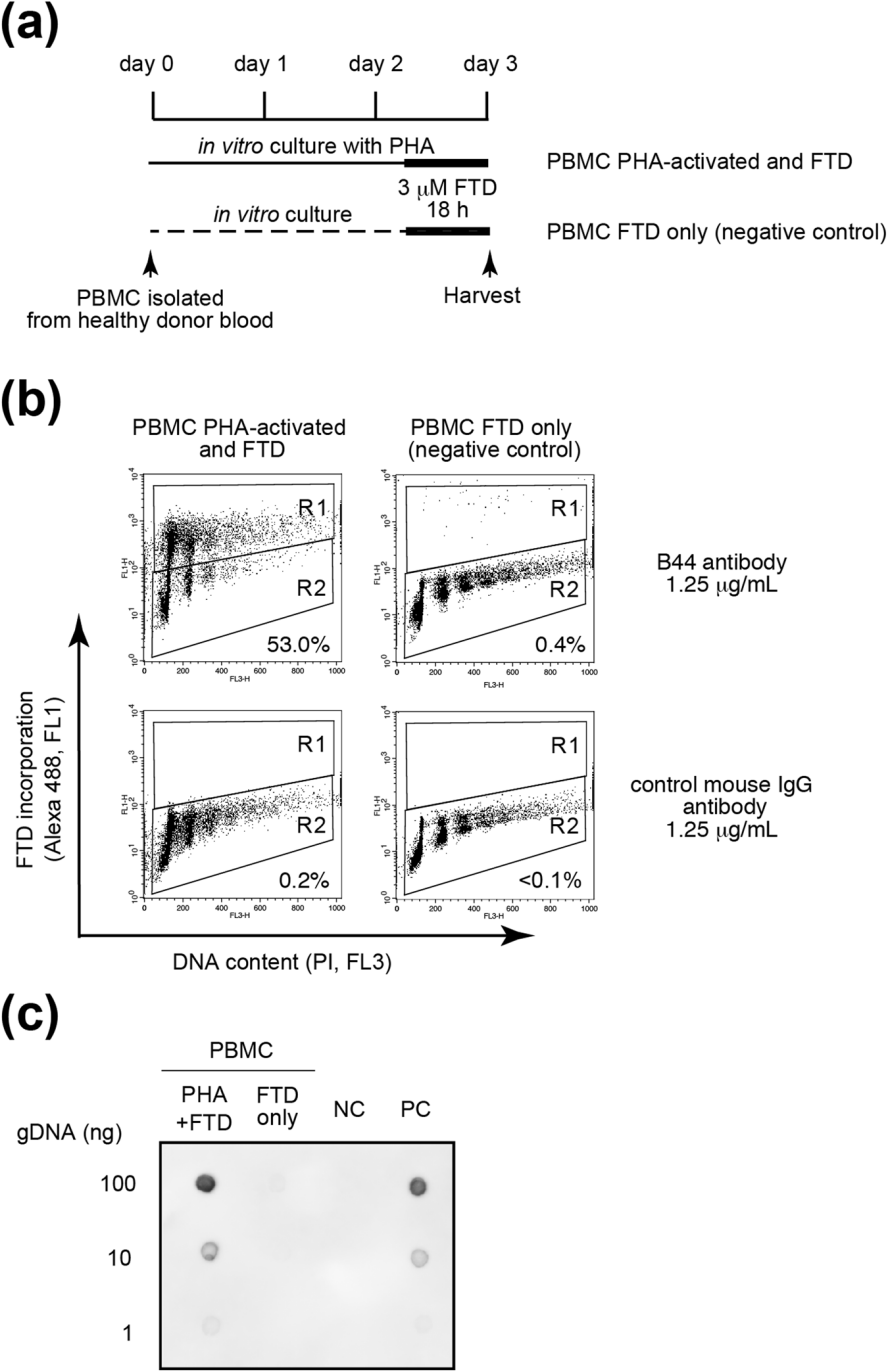
FTD incorporation into PBMCs isolated from a healthy donor and activated by PHA-P *in vitro*. (**a**) The culture schedule. PBMCs were isolated from a healthy donor, and were cultured *in vitro* with or without PHA-P for 3 days and with FTD for the last 18 hours. (**b**) Detection of FTD by FACS analysis. (**c**) Detection of FTD by dot blot analysis. NC: negative control; PC: positive control.

Our analysis of mouse bone marrow cells and spleen cells indicated that FTD is incorporated into myeloid cells as well as lymphoid cells. To determine whether FTD incorporated into DNA of PBMCs could be detected, PBMCs isolated from human healthy donor were activated with phytohaemagglutinin-P (PHA-P) and exposed to FTD (Fig. 3a). Although FTD-positive PBMCs without prior PHA-P activation could not be detected (Fig. 3b, upper right plots), more than 50% of PBMCs were FTD-positive when they were activated with PHA-P for 3 days *in vitro* (Fig. 3b, upper left plots). These results were confirmed by dot blot analysis (Fig. 3c). Intriguingly, DNA content of FTD-positive PBMCs with PHA-P activation was broad (Fig. 3b, upper left plot). This was in contrast with FTD-positive spleen cells isolated from FTD-administrated mice in which DNA content was not broad and resided at the 2 N, 4 N, or 8 N position (Fig. 2b, upper plots), suggesting that FTD-positive mouse spleen cells were in the quiescent state and not proliferating.

### The percentage of FTD-positive PBMCs isolated from metastatic colorectal cancer patients fluctuates according to the schedule of TFTD medication

**Figure 4:**
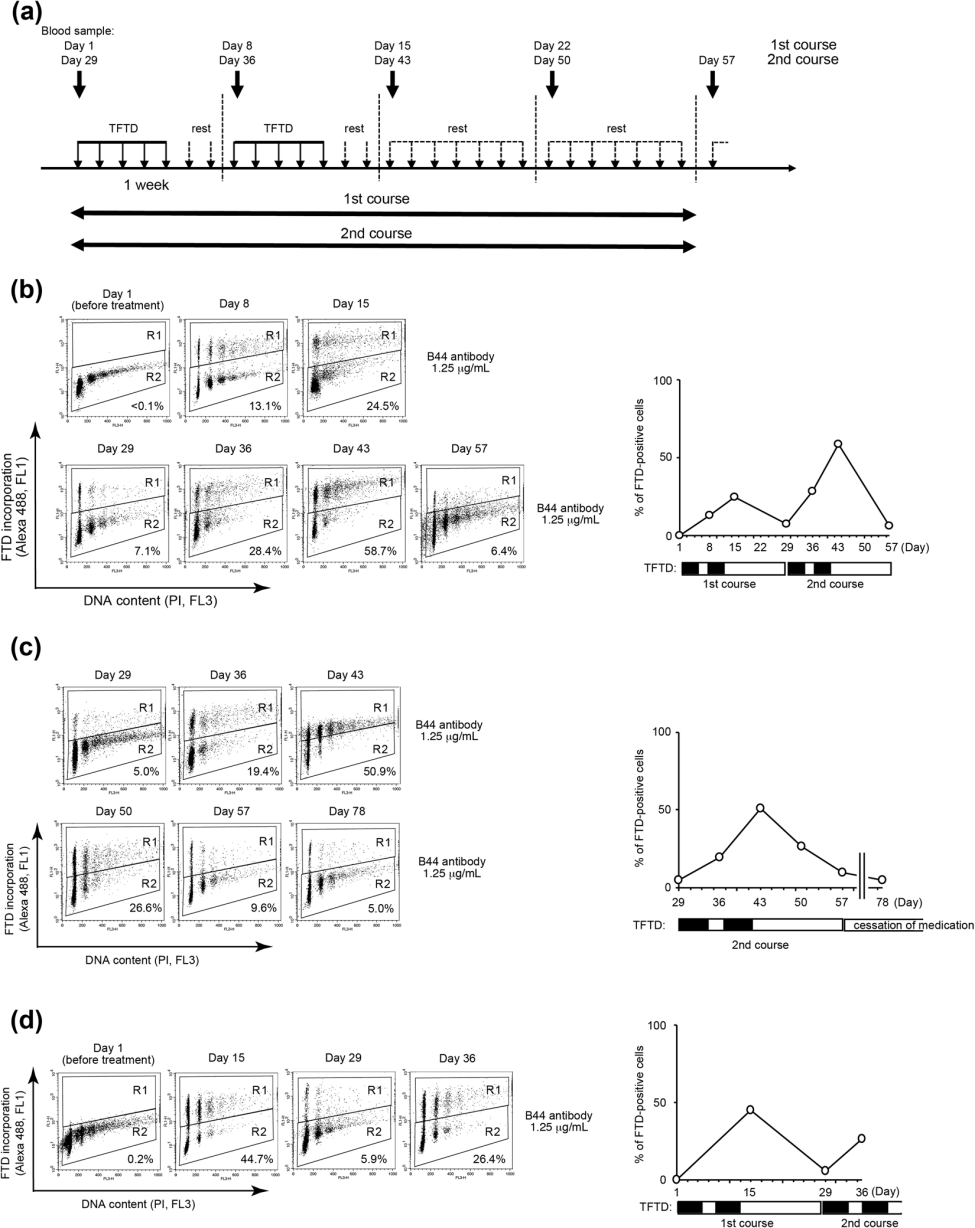
FTD incorporation into PBMCs of mCRC patients who received TFTD medication. (**a**) The schedule of TFTD medication and blood sampling (upper). The first cycle was from day 1 to day 28, and the second cycle was from day 29 to day 56. (**b**–**d**) The fluctuation of the % FTD-positive PBMCs in each mCRC patient. The schedule of TFTD medication is indicated by line bar under the graph.

Our analyses of mouse spleen cells and PHA-P activated human PBMCs *in vitro* strongly suggest that FTD should be detectable in human PBMCs from cancer patients who receive TFTD medication. To assess this, we isolated PBMCs from blood samples of three mCRC patients who received TFTD medication and analysed the FTD incorporation. The schedule of TFTD medication was as follows: twice daily after morning and evening meals, 5 continuous days a week with 2 days rest for 2 weeks, followed by 14 days cessation. mCRC patients with TFTD medication usually visit the hospital every week on the day they start TFTD medication (Fig. 4a). Very few FTD-positive PBMCs were observed before TFTD medication (Fig. 4b,d, Day 1). The percentage (%) of FTD-positive PBMCs increased during the period of TFTD medication (Fig. 4b–d, Day 1 to Day 15 and Day 29 to Day 43). The maximum % of FTD-positive PBMCs in each patient at the end of each course (Day 15 or Day 43) was various (20–60%), but the % of FTD-positive PBMCs gradually decreased to less than 10% after 2 weeks cessation (Day 29 and Day 57). These data suggest that FTD could be incorporated in the significant population of PBMCs or their progenitors during the period of TFTD administration, but the FTD-positive PBMCs in the bloodstream could be eventually diminished to the baseline level if TFTD medication is ceased for a couple of weeks.

## Discussion

To evaluate or predict the efficacy or toxicity of chemotherapy, it is important to trace the key component of the chemotherapeutic drug in clinical specimens. FTD is a key component of the novel, orally-administrated, anticancer drug, TFTD. FTD is a nucleoside analogue whose incorporation into DNA is correlated with its cytotoxicity and detected by using a specific antibody^20^. In this study, we report that FTD incorporation was detected in the bone marrow cells (Fig. 1c) and in the spleen cells of FTD-administrated mice, and show that we can monitor the fluctuation of FTD-positive lymphoid cells (Fig. 2). We also detected FTD in human PBMCs activated and exposed to FTD *in vitro* (Fig. 3). Consequently, we successfully detected FTD in PBMCs isolated from mCRC patients who received TFTD medication (Fig. 4). The current study also revealed that the proportion of FTD-positive cells in patient PBMCs fluctuated in parallel with the administration or cessation of TFTD medication. To the best of our knowledge, this is the first case to report a key component of a chemotherapeutic drug captured inside the cell of clinical specimens from cancer patients who received chemotherapy. Importantly, this method could be applied clinically and may aid the development of compound reagents that predict the efficacy or toxicity of TFTD.

FACS analysis revealed that few cells with a DNA content between 2 N and 4 N were detected when PBMCs were isolated from an mCRC patient’s blood sample (Fig. 4) or when the lymphoid cells obtained from spleen of FTD-administrated mice (Fig. 2). These cells appeared to be quiescent, either in G1 or G2 phase. In contrast, FTD-positive PBMCs activated with PHA-P *in vitro* were either in G1, S or G2 phase (Fig. 3b). FTD is incorporated into DNA during S phase^20^. FTD-positive cells were detected in the bone marrow (Fig. 1c,d), suggesting FTD can be incorporated into DNA in the myeloid cells. The FTD-positive myeloid cells in the bone marrow of patients who received TFTD medication may escape cell death, but stop proliferating, possibly because of p53-dependent sustained arrest at G2 phase^21^, differentiate into lymphoid cells and PBMCs and circulate in the bloodstream in a quiescent state. It is an intriguing question whether these FTD-positive lymphoid cells or PBMCs keep their cellular function or not.

Therapeutic drug monitoring (TDM) in the clinical specimens of patients is fundamental to optimize the effective therapeutic range. As TDM, pharmacokinetic analyses of drug in the bloodstream or urine based on immunoassay, *e.g*. enzyme-linked immunosolvent assay, are the standard methods for clinical use^22,23^. High pressure liquid chromatography (HPLC) and liquid chromatography-mass spectrometry (LC-MS)-based TDM methods are also employed if the immunoassay-based methods are not applicable. Recently, MS-based imaging methodologies, *i.e*. a matrix-assisted laser desorption/ionization imaging MS^24^, have been developed, but their clinical application has not necessarily been achieved. Our immunodetection method of FTD is simple and easy, and provides a direct evidence that the key component of a chemotherapeutic drug reaches and affects the target cells, since it was already shown that FTD incorporation into DNA of proliferating cells or xenografts is significantly correlated with its cytotoxicity^12,18^. The clinical application of this method could be achievable and provide a good predictive biomarker of the efficacy or adverse effects of TFTD medication.

One possible clinical application of the FTD-detection method in PBMCs is to predict the conspicuous adverse effects of TFTD, such as neutropenia. As mentioned above, TFTD significantly improved the OS and PFS of patients with mCRC, but the disease control rate of TFTD-treated patients in the RECOURSE trial was only 44%^10^. From the analysis of patients who received TFTD medication in the RECOURSE trial, the occurrence rate of Grade 3/4 neutropenia was significantly associated with the prognosis of patients who received TFTD medication^14^. Kasi *et al*. also reported that neutropenia caused by TFTD during the first cycle was associated with better efficacy^15^. However, neither the area under the blood concentration-time curve nor the maximum concentration of TFTD was associated with the occurrence rate of Grade 3/4 neutropenia^14^. As FTD displays the effect via its incorporation into DNA, the proportion of the FTD-positive PBMCs might be correlated with the grade of neutropenia.

In conclusion, the detection method of FTD might enable the evaluation or prediction of the efficacy or adverse effect of TFTD medication, because this method provides direct and demonstrable evidence that the key component of a chemotherapeutic drug is present in the tissues of target. Indeed, our quantitative analysis of FTD incorporation in PBMCs from mCRC patients who received TFTD medication revealed that the proportion of FTD-positive PBMCs fluctuated according to the schedule of TFTD medication. However, we cannot evaluate the clinical values of this method because clinical data of these patients have not been obtained yet. This is one of the limitations of our current study. Further prospective analyses regarding the association of this finding with the clinical data, including the response rate or the frequency of adverse effects, are necessary. In future, the clinical application of this method may contribute to improve the use of this valuable chemotherapeutic drug and to encourage the development of companion diagnostic reagents.

## Materials and Methods

### Animals

Five-week-old BALB/cAJcl-*nu*/*nu* mice and six-week-old male BALB/cAJcl mice were purchased from CLEA Japan, Inc. (Tokyo, Japan). All animal care and experimental procedures were approved by the committee on the Ethics of Animal Experiments, Kyushu University, and were conducted in accordance with the Guidelines for Animal Experiments of Kyushu University and national standards.

### Xenograft model

BALB/cAJcl-*nu/nu* mice were implanted with HCT-116 (1 × 10^7^ cells). After 10 days, FTD (50 mg/kg) was injected intraperitoneally.

### Mouse spleen cell preparation

BALB/cAJcl mice (N = 4) were given FTD orally (50 mg/kg/day, b.i.d.) for 3 days, and their spleens were removed after cervical dislocation under anaesthesia 1, 3, 7, and 14 days after the last administration of FTD. Spleen cells were isolated from single cell suspension of spleens by centrifugation on Lympholyte-M^®^ (Cederlane Inc., Burlington, Ontario, Canada).

### mCRC patients

We analysed three mCRC patients who received TFTD medication after first/second/third line treatment including fluoropyrimidine, oxaliplatin, irinotecan and a molecular targeted drug (Bevacizumab, Cetuximab or Panitumumab) at Kyushu University Hospital. mCRC patients were administrated TFTD twice daily after morning and evening meals, 5 continuous days a week with 2 days rest for 2 weeks, followed by 14 days cessation. The treatment was repeated every 28 days. The administration was stopped or skipped dependent upon the patient physical condition. Blood samples were obtained on days 1, 8 and 15 of the course. Written informed consent was obtained from all patients. The institutional review board of Kyushu University approved this study (#28–46). The informed consent was obtained from all participants, and the authors declare that all experiments were performed in accordance with relevant guidelines and regulations.

### Immunohistochemistry

Bone marrow and xenografts obtained from FTD-administrated mice were fixed with 4% paraformaldehyde in PBS buffer for 24 hours and embedded into paraffin. To obtain the bone marrow, fixed bone was demineralized using demineralization solution B for 3 days (Cat#041-22031, Wako Pure Chemical Industries, Ltd., Osaka, Japan). Immunohistochemical staining of FTD in paraffin-embedded specimens was performed using a BrdU *in situ* detection kit (BD Bio science, Cat#550803) as described previously^20^.

### Isolation of PBMCs from blood samples

Blood samples obtained from a healthy donor and mCRC patients were collected in tubes containing anticoagulant solution, heparin and citrate phosphate dextrose (NP-SC1000, Cat#31–620, Nipro, Osaka, Japan), respectively. The PBMCs were then isolated by centrifugation on Lymphoprep™ (Cat#1114544, CosmoBio, Tokyo, Japan).

### FACS analysis

Mouse spleen cells and human PBMCs were fixed with 70% ethanol. The fixed samples were acid depurinated with 2 N HCl for 30 minutes at room temperature, incubated with anti-BrdU antibodies (B44 (Cat#347580, BD Biosciences, San Jose, CA)) at 1.25 µg/ml, stained with Alexa 488-conjugated secondary antibodies (Thermo Fisher Scientific, Waltham, MA) and counterstained with propidium iodide. The prepared samples were analysed by FACSCalibur (BD Biosciences, San Jose, CA). A negative control of FTD staining was prepared from each sample incubated with control mouse IgG antibody at 1.25 µg/ml. The boundary of the regions of FTD-positive (R1) and –negative (R2) cells in 2-dimension dot plot was determined by the negative control in each sample. The percentage of FTD-positive cells was calculated by dividing the cell number in the R1 region with the total cell number (the cell number in the R1 and R2 regions).

### Dot blot analysis of genomic DNA

Genomic DNA was purified from mouse spleen cells, human PBMCs and a human cancer cell line using the Gentra Puregene Cell kit (Qiagen GmbH, Hilden, Germany). Dot blot experiments were performed as described previously^20^.

### Availability of materials and data

The materials, data and associated protocols are available to readers without any restrictions on the availability of materials or information.

### Ethical approval

As described in Materials and Methods, the institutional review board of Kyushu University approved this study (#28–46).

## Electronic supplementary material

Dataset 1
