# Supplementary material for: Monitoring trifluridine incorporation in the peripheral blood mononuclear cells of colorectal cancer patients under trifluridine/tipiracil medication
Source: Sci Rep. 2017 Dec 5;7:16969. doi: 10.1038/s41598-017-17282-5 (PMC5717244; doi:10.1038/s41598-017-17282-5)
Supplement: Supplementary file 1 — Dataset 1 [file 41598_2017_17282_MOESM1_ESM.doc]

**Supplementary Information for**

**Monitoring trifluridine incorporation in the peripheral blood mononuclear cells of colorectal cancer patients under trifluridine/tipiracil medication.**

Ryota Nakanishi^1^, Hiroyuki Kitao^2,3,*^, Mamoru Kiniwa^3,4^, Yosuke Morodomi^1^, Makoto Iimori^2^, Junji Kurashige^1^, Masahiko Sugiyama^1^, Yuichiro Nakashima^1^, Hiroshi Saeki^1^, Eiji Oki^1^, Yoshihiko Maehara^1^

^1^Department of Surgery and Science, Graduate School of Medical Sciences, ^2^Department of Molecular Cancer Biology, Graduate School of Pharmaceutical Sciences, ^3^Innovative Anticancer Strategy for Therapeutics and Diagnosis Group, Innovation Center for Medical Redox Navigation, Kyushu University, Fukuoka, Japan, ^4^Taiho Pharmaceutical Co. Ltd., Tokushima and Ibaraki, Japan.

Corresponding author: Hiroyuki Kitao, PhD

Department of Molecular Cancer Biology, Graduate School of Pharmaceutical Sciences, Kyushu University, Maidashi 3-1-1, Higashi-ku, Fukuoka 812-8582, Japan. Tel: +81-92-642-6499; Fax: +81-97-642-5482; E-mail: [hkitao@phar.kyushu-u.ac.jp](mailto:hkitao@phar.kyushu-u.ac.jp)


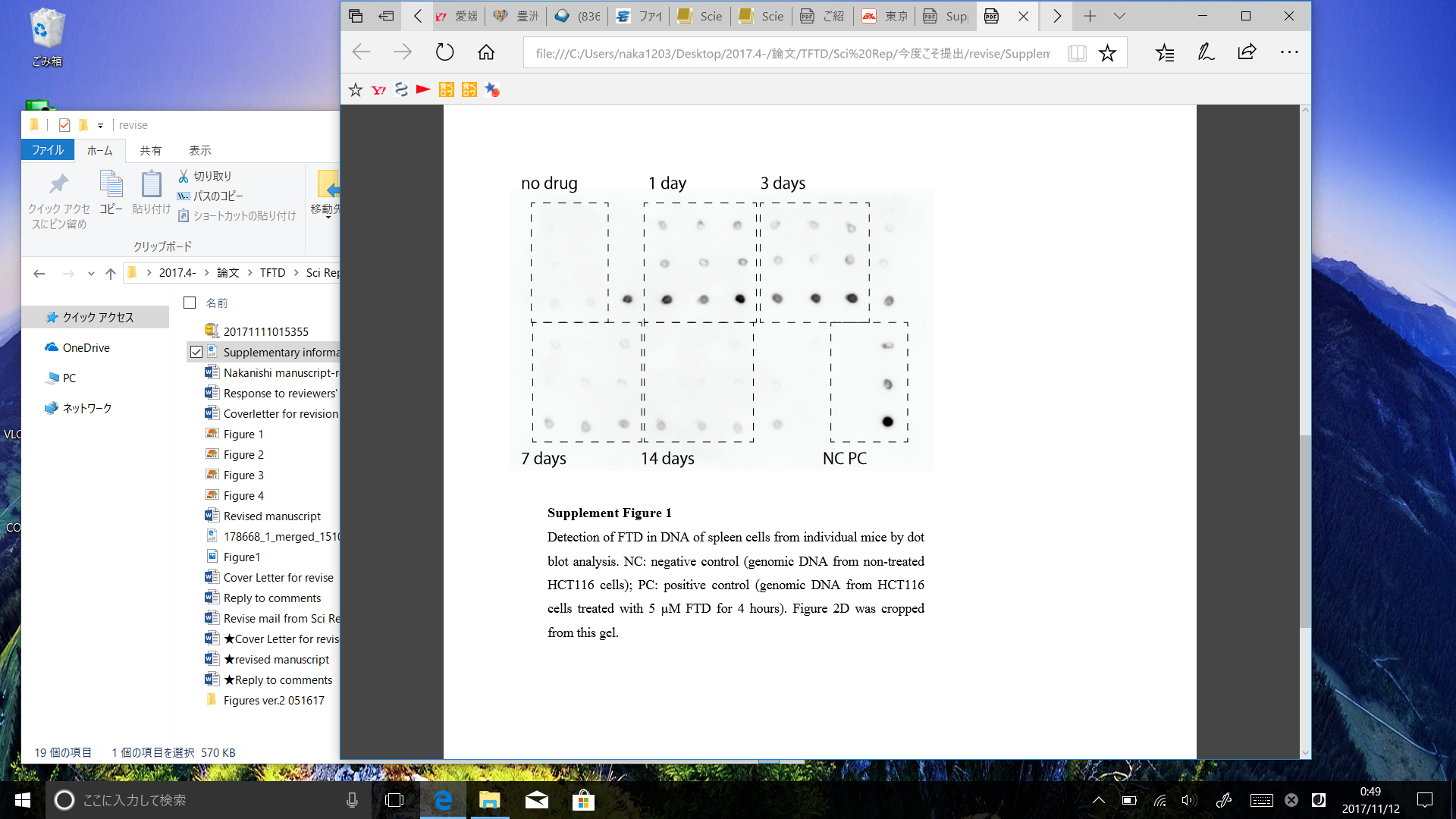


Supplement Figure1

Detection of FTD in DNA of spleen cells fromindividual mice by dot blot analysis. NC: negative control (genomic DNA from non-treated HCT116 cells); PC: positive control (genomic DNA from HCT116 cells treated with 5 µM FTD for 4 hours). Figure 2D was cropped fromthisgel.
